# Dataset of microplastics in the mangrove brachyuran crabs at Setiu Wetlands, Peninsular Malaysia

**DOI:** 10.1016/j.dib.2023.109420

**Published:** 2023-07-18

**Authors:** Nur Hannah Abd Rahim, Behara Satyanarayana, Yusof Shuaib Ibrahim, Christelle Not, Izwandy Idris, Jarina Mohd Jani, Stefano Cannicci, Farid Dahdouh-Guebas

**Affiliations:** a Mangrove Research Unit (MARU), Institute of Oceanography and Environment (INOS), Universiti Malaysia Terengganu (UMT), Kuala Nerus 21300, Malaysia; b Systems Ecology and Resource Management Research Unit (SERM), Université Libre de Bruxelles-ULB, 1050 Brussels, Belgium; c Mangrove Specialist Group (MSG), Species Survival Commission (SSC), International Union for the Conservation of Nature (IUCN), c/o Zoological Society of London, London, United Kingdom; d Microplastic Research Interest Group (MRIG), Faculty of Science and Marine Environment, Universiti Malaysia Terengganu (UMT), Kuala Nerus 21300, Malaysia; e Environmental Geochemistry & Oceanography Research Group, Department of Earth Sciences,The University of Hong Kong, Hong Kong SAR; f Swire Institute for Marine Science, The University of Hong Kong, Hong Kong SAR; g South China Sea Repository and Reference Centre, Institute of Oceanography and Environment (INOS), Universiti Malaysia Terengganu (UMT), Kuala Nerus 21300, Malaysia; h Biodiversity Conservation and Management Program, Faculty of Science and Marine Environment, Universiti Malaysia Terengganu (UMT), Kuala Nerus 21300, Malaysia; i Department of Biology, University of Florence, 50019 Florence, Italy; j Ecology & Biodiversity Research Unit, Department of Biology, Vrije Universiteit Brussel-VUB, 1050 Brussels, Belgium

**Keywords:** Plastic waste, Pollution, Macrobenthos, Feeding habit

## Abstract

The present dataset provides information on the abundance of microplastics (MPs) in relation to different feeding habits of the four mangrove brachyuran crab species namely*, Parasesarma eumolpe, Austruca annulipes, Metaplax elegans* and *Scylla olivacea* at Setiu Wetlands in Peninsular Malaysia. Three sites namely, Pulau Layat (upstream, close to the core mangrove zone), Kampung Pengkalan Gelap (midstream, close to the settlements), and Pulau Sutung (downstream, close to the shifted river mouth) were chosen for the sample collection (through hand catch method and traps) in both the dry (Feb-Mar 2021) and the wet (Dec 2021 - Jan 2022) seasons. The cardiac stomach of each crab was dissected, digested in potassium hydroxide and then filtered through a 1.6 µm pore size glass fibre filter using the vacuum pump. The abundance, type and colour of MPs per crab individual were determined under a stereomicroscope (Carl Zeiss Stemi 508, China) attached to the digital camera (Axiocam 208 colour). The general abundance of MPs was found in the order of carnivorous *S. olivacea* > microphytobenthos feeder *A. annulipes* > herbivorous *P. eumolpe* > detritivorous *M. elegans*. The data also reveal morphometric measurements such as body weight, gut weight, carapace width and carapace length of the crab specimens. The information given in this article is useful for study replications and scientific comparisons, especially with brachyuran crabs and other organisms with similar feeding guilds, in the mangroves of Malaysia and elsewhere.

**Specifications Table**

**Table:** 

Subject	Environmental pollution
Specific subject area	Microplastics (MPs) ingestion in mangrove brachyuran crabs at Setiu Wetlands, Peninsular Malaysia
Type of data	Figure and table
How the data were acquired	Hand catch method and traps, stereomicroscope (Carl Zeiss Stemi 508, China), digital camera (Axiocam 208 colour)
Data format	Raw
Description of data collection	Samples of the four brachyuran crab species (n= 233) with different feeding habits were collected from three sites during the dry (Feb-Mar 2021) and the wet (Dec 2021-Jan 2022) seasonal periods. While *Parasesama eumolpe* (found in the vegetated areas), *Austruca annulipes* (found in the sandy waterfronts), and *Metaplax elegans* (found on the exposed mud) were captured by hand, *Scylla olivacea* (found in the waterfront and/or *Rhizopora* roots) was collected by using the traps called “bubu”. All crab samples were kept in an aluminium container and transferred to the laboratory on the same day of collection. The specimens were then rinsed with ultra-pure deionised water (MiliQ), weighed, and measured for its carapace width and length, before preserving them at -18 ˚C. Further processing of the samples involved dissection, chemical digestion of the stomachs (using potassium hydroxide), and filtration (using 1.6 µm pore size glass fibre filter paper). Quantification of the MPs on each filter paper was done under a stereomicroscope attached to the digital camera.
Data source location	Institution: Institute of Oceanography and Environment, Universiti Malaysia Terengganu City/Town/Region: Setiu Wetlands, Terengganu, East coast of Peninsular Malaysia Country: Malaysia Latitude and longitude: 05° 36.'30”- 05° 42.'30” N, 102° 40.'30” - 102° 48.'30” E
Data accessibility	Data could be found with this article and online at https://data.mendeley.com/datasets/z2ffhznyj6/3 DOI:10.17632/z2ffhznyj6.1

## Value of the Data

• Studies on the ingestion of MPs by brachyuran crabs are limited and thus help to fulfill the knowledge gaps in relevance.
• The data could be used as baseline information for further research in Malaysia
• Brachyuran crabs as indicator species for plastic pollution in mangrove wetlands.

## 1 Objective

To analyse the abundance/composition of MPs in the mangrove crabs with different feeding behaviours at Setiu Wetlands. This dataset also helps to assess the variations in the MPs at different sampling sites for the dry and wet seasonal periods.

## 2 Data Description

**Fig. 1:**
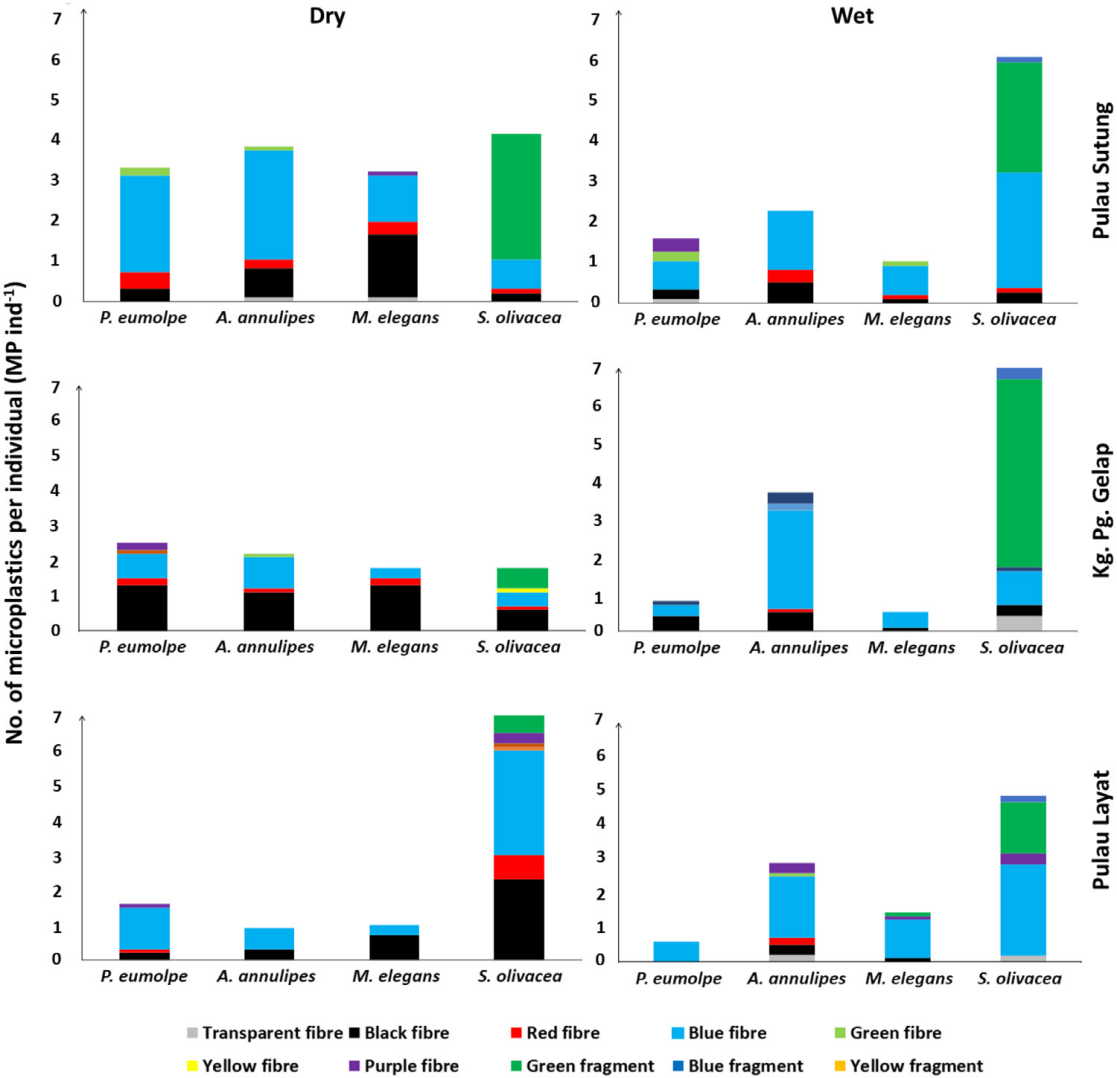
Number of microplastics in different brachyuran crab species across three sampling sites at the Setiu Wetlands for dry and wet seasonal periods (Kg. Pg. Gelap = Kampung Pengkalan Gelap) (colour of the bar charts refer to the colour of the MPs found).

The raw data file, “Dataset of microplastics in the mangrove brachyuran crabs at Setiu Wetlands, Peninsular Malaysia” with the five Excel spreadsheets could be retrieved from the Mendeley Data repository. About 79% of the total (233) crab samples were identified with MPs. The first two spreadsheets, named “MPs_DryPeriod” and “MPs_WetPeriod” show the abundance of MPs (size: 0.01 - 5 mm) in *P. eumolpe, A. annulipes, M. elegans* and *S. olivacea* across three sampling sites for the dry (Feb-Mar 2021) and wet (Dec 2021 - Jan 2022) seasons, respectively. The total number of MPs, represented by different coloured fibre and fragment types (such as blue, black, red, green, etc.), is given for each crab replicate. The data from these two spreadsheets were summarized and presented in Fig. 1. The next two spreadsheets named “Crab_DryPeriod” and “Crab_WetPeriod” show the morphometric measurements of those four crab species at the three sampling sites for both the dry and the wet seasons separately. These measurements include sex, body weight, gut weight, carapace width and carapace length of each crab replicate. Site- and season-based variations in the range of morphometric measurements are provided in Tables 1 and 2. The final spreadsheet contains the GPS coordinates of each sampling site with corresponding locational descriptions (Table 3).

**Table 1:** The range of morphometric measurements of the brachyuran crabs obtained from three sites during the dry season (Feb-Mar 2021) at Setiu Wetlands (*P. eumolpe = Parasesarma eumolpe; A. annulipes = Austruca annulipes; M. elegans = Metaplax elegans; S. olivacea = Scylla olivacea*) (Kg. Pg. Gelap = Kampung Pengkalan Gelap).

Site	Crab species	Sex	Crab weight (g)	Gut weight (g)	Carapace width (cm)	Carapace length (cm)
Pulau Layat	*P. eumolpe*	Male	1.63 - 5.09	0.05 - 0.24	14.89 - 20.23	12.19 - 17.37
	*P. eumolpe*	Female	1.21–3.85	0.06–0.20	13.75–20.34	11.11–16.86
	*A. annulipes*	Male	0.34–1.00	0.01–0.03	10.78–14.26	6.30–8.81
	*A. annulipes*	Female	0.30–0.47	0.06–0.20	11.16–13.15	6.59–8.08
	*M. elegans*	Male	0.47–0.94	0.02	10.86–13.69	7.41–9.05
	*M. elegans*	Female	0.19–0.93	0.01–0.03	8.44–14.54	6.18–10.02
	*S. olivacea*	Male	93.11–158.38	0.99–1.44	79.05–95.88	53.65–63.96
	*S. olivacea*	Female	77.75–106.03	0.78–1.78	74.37–83.16	49.37–57.31
Kg. Pg. Gelap	*P. eumolpe*	Male	0.78–3.67	0.04–0.12	11.82–18.58	9.72–15.03
	*P. eumolpe*	Female	0.78–2.18	0.05–0.11	11.87–17.50	9.70–13.83
	*A. annulipes*	Male	0.33–0.77	0.01	9.86–16.49	5.92–7.53
	*A. annulipes*	Female	0.10–0.87	0.01–0.03	7.66–13.58	4.71–8.23
	*M. elegans*	Male	0.42–1.33	0.01–0.03	10.53–14.53	7.49–9.55
	*M. elegans*	Female	0.11–0.55	0.01	8.17–11.91	5.89–8.27
	*S. olivacea*	Male	64.16–82.64	0.60–0.84	73.05–76.04	49.92–52.33
	*S. olivacea*	Female	76.24–110.49	0.88–1.31	74.51–86.94	50.96–58.35
Pulau Sutung	*P. eumolpe*	Male	0.76–5.72	0.05–0.26	12.44–23.72	10.63–18.44
	*P. eumolpe*	Female	0.37–1.74	0.03–0.09	9.29 -15.49	7.51–12.54
	*A. annulipes*	Male	0.72–0.95	0.02	12.58–14.28	7.33–8.56
	*A. annulipes*	Female	0.13–0.19	0.01	7.75–8.84	4.96–5.36
	*M. elegans*	Male	0.37–0.76	0.01–0.02	9.85–12.09	7.04–8.38
	*M. elegans*	Female	0.45–0.70	0.02–0.03	10.94–13.43	7.76–9.36
	*S. olivacea*	Male	102.69–128.99	1.00–1.47	80.13–90.01	53.19–60.22
	*S. olivacea*	Female	53.25–124.01	0.62–2.12	68.26–90.34	46.28–61.64

**Table 2:** The range of morphometric measurements of the brachyuran crabs obtained from three sites during the wet season (Dec 2021-Jan 2022) at Setiu Wetlands (*P. eumolpe = Parasesarma eumolpe; A. annulipes = Austruca annulipes; M. elegans = Metaplax elegans; S. olivacea = Scylla olivacea*) (Kg. Pg. Gelap = Kampung Pengkalan Gelap).

Site	Crab species	Sex	Crab weight (g)	Gut weight (g)	Carapace width (cm)	Carapace length (cm)
Pulau Layat	*P. eumolpe*	Male	1.82–4.14	0.11–10.24	1.51–1.97	1.21–1.58
	*P. eumolpe*	Female	0.27–2.43	0.06–1.17	1.62–1.73	1.28–1.42
	*A. annulipes*	Male	0.76–1.35	0.02–0.06	1.40–1.72	0.81–1.23
	*A. annulipes*	Female	0.11–0.78	0.01–0.06	0.85–1.45	0.50–0.95
	*M. elegans*	Male	1.01–1.35	0.01–0.04	1.42–1.47	0.92–1.00
	*M. elegans*	Female	0.73–1.25	0.03–0.06	1.32–1.55	0.90–1.06
	*S. olivacea*	Male	40.09–296.19	0.58–1.80	7.78–11.59	4.99–7.88
	*S. olivacea*	Female	69.36–161.15	0.52–1.33	8.52–10.60	6.04–7.31
Kg. Pg. Gelap	*P. eumolpe*	Male	1.26–3.24	0.08–0.16	1.40–1.83	1.22–1.48
	*P. eumolpe*	Female	0.97–1.55	0.06–0.12	1.28–1.54	1.02–1.22
	*A. annulipes*	Male	0.66–1.08	0.03–0.09	1.43–1.84	0.72–0.96
	*A. annulipes*	Female	0.34–0.69	0.01–0.05	1.20–1.59	0.77–0.91
	*M. elegans*	Male	0.57–1.51	0.03–0.36	1.17–1.68	0.70–1.05
	*M. elegans*	Female	0.45–1.29	0.02–0.07	0.85–1.65	0.50–1.12
	*S. olivacea*	Male	103.25–162.90	0.77–1.47	8.25–10.21	5.65–6.98
	*S. olivacea*	Female	134.73–208.55	0.91–1.77	8.76–11.44	6.57–8.97
Pulau Sutung	*P. eumolpe*	Male	1.20–2.47	0.09–0.22	1.39–1.90	1.11–1.60
	*P. eumolpe*	Female	0.74–2.83	0.06–0.24	1.28–1.94	1.00–1.51
	*A. annulipes*	Male	0.58–1.17	0.02–0.03	1.21–1.58	0.73–0.95
	*A. annulipes*	Female	0.21–0.39	0.02–0.04	1.04–1.30	0.61–0.74
	*M. elegans*	Male	0.31–0.49	0.01–0.05	1.02–1.09	0.70–0.84
	*M. elegans*	Female	0.29–0.57	0.01–0.05	0.95–1.27	0.68–0.86
	*S. olivacea*	Male	64.84–154.23	0.38–2.47	8.24–9.79	5.42–6.77
	*S. olivacea*	Female	156.00	1.17	10.1	7.17

**Table 3:** Coordinates and descriptions of the sampling sites.

Station	Latitude (°N)	Longitude (°E)	Notes
Pulau Layat	5.699367°	102.693017°	Upstream (mangrove core zone)
Kampung Pengkalan Gelap	5.678450°	102.712433°	Located near settlements, ecotourism boardwalk and aquaculture site
Pulau Sutung	5.653861°	102.751917°	Downstream (close to shifted river mouth)

## 3 Experimental Design, Materials and Methods

### 3.1 Study area and sampling sites

Setiu Wetlands, connected to the South China Sea on the East Coast of Peninsular Malaysia, are strongly influenced by the daily flood/ebb conditions. The seasonal changes have a profound impact on the hydrodynamics in the lagoon [1]. Three sampling sites namely, Pulau Layat (close to the core mangrove i.e., the largest patch with rich species diversity), Kampung Pengkalan Gelap (between the core mangrove and the shifted river mouth), and Pulau Sutung (close to the shifted river mouth) were chosen to evaluate the possible use of the mangrove brachyuran crabs as indicators for MPs contamination.

### 3.2 Collection of crabs

Four brachyuran crab species with distinct feeding modes, namely the herbivorous *Parasesarma eumolpe* (De Man, 1895) (Sesarmidae), the detritivorous *Metaplax elegans* De Man, 1888 (Varunidae), the microphytobenthos feeder *Austruca annulipes* (Milne-Edwards, 1837) (Ocypodidae), and the carnivorous *Scylla olivacea* (Herbst, 1796) (Portunidae) were collected from all three sampling sites covering the dry (Feb-Mar 2021) and the wet (Dec 2021-Jan 2022) seasonal periods.

Except *S. olivacea*, all specimens were collected by hand catch method during the low tide [2]. For *S. olivacea*, a traditional trap called “bubu” was placed close to the waterfront and/or *Rhizophora* roots. A total of ten individuals per species was targeted from each sampling site per season. However, the lower catch of some individuals in the wet period, especially *S. olivacea*, limited the sample size to 233 (seven specimens less than the target). The crab samples were transferred to the laboratory on the same day of collection, rinsed with ultra-pure deionized water (MiliQ), weighed and measured its carapace width and length, before preserving them in a glass beaker (covered with aluminium foil) at -18 ˚C until further processing.

### 3.3 Sample preparation and data analyses

The dissected stomach of each crab was first weighed and then digested with potassium hydroxide in a glass vial, covered by aluminium foil. The vials were placed in an oscillation water bath at 60 ˚C for 48-72 hours at 240 rpm. The digested solution was filtered through a glass filter paper (1.6 µm pore size) by using the vacuum pump and dried in a desiccator for at least 24 hours. The number of MPs left on the filter paper were carefully observed, identified, grouped, counted, and measured under a stereomicroscope (Carl Zeiss Stemi 508, China) attached to the digital camera (Axiocam 208 colour). The MPs were also categorized into bead, fibre, fragment, and pellet types along with their colour variations [3]. The hot needle test was used to separate the MPs from the other particles [4].

### 3.4 Contamination control

All lab apparatus were cleaned with the filtered ethanol and ultra-pure MiliQ deionized water. Other precautionary measures included conducting the experiments in a closed and clean chamber, avoiding unnecessary opening of the filtration setup, covering the petri dishes and other containers with a aluminium foil, wearing the nitrile rubber gloves and a cotton lab coat. Furthermore, the procedural blanks, without biological tissue, were tested to assess the background contamination.

## Ethics Statements

No ethics approval is required for this study in accordance with the U.K. Animals (Scientific Procedures) Act, 1986 as the brachyuran crabs are invertebrates and not listed as protected species. However, the permission to conduct this study was obtained from the Management Council of Terengganu State Park, the governing body for Setiu Wetlands.

## CRediT authorship contribution statement

**Nur Hannah Abd Rahim:** Visualization, Methodology, Investigation, Writing – original draft. **Behara Satyanarayana:** Supervision, Conceptualization, Methodology, Writing – review & editing, Funding acquisition. **Yusof Shuaib Ibrahim:** Methodology, Resources, Validation. **Christelle Not:** Methodology. **Izwandy Idris:** Conceptualization, Writing – review & editing. **Jarina Mohd Jani:** Writing – review & editing. **Stefano Cannicci:** Methodology, Conceptualization, Visualization, Writing – review & editing. **Farid Dahdouh-Guebas:** Methodology, Conceptualization, Writing – review & editing.

## Declaration of Competing Interest

The authors declare that they have no known competing financial interests or personal relationships that could have appeared to influence the work reported in this paper.

## Data Availability

Dataset of microplastics in the mangrove brachyuran crabs at Setiu Wetlands, Peninsular Malaysia (Original data) (Mendeley Data).
